# Autonomous feedback loop of RUNX1-p53-CBFB in acute myeloid leukemia cells

**DOI:** 10.1038/s41598-017-16799-z

**Published:** 2017-11-30

**Authors:** Ken Morita, Mina Noura, Chieko Tokushige, Shintaro Maeda, Hiroki Kiyose, Gengo Kashiwazaki, Junichi Taniguchi, Toshikazu Bando, Kenichi Yoshida, Toshifumi Ozaki, Hidemasa Matsuo, Seishi Ogawa, Pu Paul Liu, Tatsutoshi Nakahata, Hiroshi Sugiyama, Souichi Adachi, Yasuhiko Kamikubo

**Affiliations:** 1 Department of Human Health Sciences, Graduate School of Medicine, Kyoto University, Sakyo-ku, Kyoto, 606-8507 Japan; 2 Department of Chemistry, Graduate School of Science, Kyoto University, Sakyo-ku, Kyoto, 606-8502 Japan; 3 Department of Pathology and Tumor biology, Kyoto University, Sakyo-ku, Kyoto, 606-8315 Japan; 4 Laboratory of DNA Damage Signaling, Chiba Cancer Center Research Institute, Chuo-ku, Chiba, 260-8717 Japan; 5 Oncogenesis and Development Section, National Human Genome Research Institute, National Institutes of Health, Bethesda, MD 20892 USA; 6 Drug Discovery Technology Development Office, Center for iPS cell research and application (CiRA), Kyoto University, Sakyo-ku, Kyoto, 606-8507 Japan; 7 Department of Pediatrics, Graduate School of Medicine, Kyoto University, Sakyo-ku, Kyoto, 606-8507 Japan

## Abstract

Although runt-related transcription factor 1 (RUNX1) and its associating core binding factor-β (CBFB) play pivotal roles in leukemogenesis, and inhibition of RUNX1 has now been widely recognized as a novel strategy for anti-leukemic therapies, it has been elusive how leukemic cells could acquire the serious resistance against RUNX1-inhibition therapies and also whether CBFB could participate in this process. Here, we show evidence that p53 (TP53) and CBFB are sequentially up-regulated in response to *RUNX1* depletion, and their mutual interaction causes the physiological resistance against chemotherapy for acute myeloid leukemia (AML) cells. Mechanistically, p53 induced by *RUNX1* gene silencing directly binds to *CBFB* promoter and stimulates its transcription as well as its translation, which in turn acts as a platform for the stabilization of RUNX1, thereby creating a compensative RUNX1-p53-CBFB feedback loop. Indeed, AML cells derived from relapsed cases exhibited higher *CBFB* expression levels compared to those from primary AML cells at diagnosis, and these *CBFB* expressions were positively correlated to those of p53. Our present results underscore the importance of RUNX1-p53-CBFB regulatory loop in the development and/or maintenance of AML cells, which could be targeted at any sides of this triangle in strategizing anti-leukemia therapies.

## Introduction

CBFB is the beta subunit of heterodimeric core-binding transcription factor which master-regulates vital subsets of genes implicated in hematopoiesis and osteogenesis^1^. This beta subunit which lacks DNA-binding capability, facilitates the association of DNA-binding runt domain in alpha subunit with its target DNA sequences (5′-TGTGGT-3′ and much rarely 5′-TGCGGT-3′) in various gene promoters as well as enhancers^2^. The alpha subunit is constituted of three representative members; RUNX1, RUNX2 and RUNX3. Although each of RUNX family members plays distinct physiological roles *in vivo*, their functions are consistently redundant in malignant cells and thus could be targeted simultaneously for anti-tumor strategies^3,4^. We have recently reported that anti-tumor potential of RUNX inhibition is primarily mediated by pro-apoptotic p53-dependent cell death pathway^5^. Based on our results, RUNX inhibition led to the transcriptional down-regulation of genes involved in the prohibition of p53 such as BCL11A and TRIM24, resulting in up-regulation of p53-mediated pro-apoptotic signaling in tumor cells. In addition, we have also demonstrated that the intracellular amount of CBFB which is equivalent to that of total RUNX (RUNX1 + RUNX2 + RUNX3), is consistently higher in malignant tissues relative to their corresponding normal ones, suggesting that CBFB is one of the ideal targets for anti-cancer therapies^5^.

Tumor suppressor p53, a distant relative of RUNX family^6^, is widely known as a nuclear transcription factor that regulates the expression of stress response genes and mediates a variety of anti-proliferative processes through transactivating its downstream target genes implicated in cell cycle checkpoints, DNA damage/repair and apoptosis^7^. The p53-resposive element has been extensively studied and the most reliable sequences are currently characterized as 10-nucleotide half-sites of RRRCWWGYYY-N-RRRCWWGYYY (R = purine, Y = pyrimidine, W = A/T and N = 0 to 13-nucleotide spacer)^8^. Of note, the emerging evidence indicates that the potential p53-target sites are not always restricted to the above-mentioned original decamer half-sites, and DNA sequences with several nucleotide mismatches seems acceptable and functional^9–11^, implying that number of p53-target genes might be much greater than that previously estimated. Consistent with its extreme importance as an oncosuppressor, somatic mutations of *p53* gene have been considered to be one of the most frequent alterations in human cancers, and most mutations are single-base substitutions found within the genomic region encoding its sequence-specific DNA-binding domain^12,13^. In a sharp contrast to wild-type p53 with the extremely short half-life, mutated p53 acquires oncogenic gain-of-function properties with the extended half-life and acts as a dominant-negative inhibitor against wild-type p53^14,15^. Since *p53* mutations are detectable primarily within its central DNA-binding domain, it is highly likely that mutant p53 lacks sequence-specific transactivation ability or acquires a capability to induce certain set of its target genes distinct from that of wild-type p53^13^.

In contrast to the majority of tumors, it has been described that *p53* is infrequently mutated in overall *de novo* AML cases (less than 10%)^16^. It is worth noting, however, that its mutation rate elevates strikingly in complex karyotype *de novo* AML cases^17,18^ or therapy-related AML cases and they display a poor prognosis^19^. Wong TN *et al*. have recently described that *p53* mutations arise during the quite early phase of the disease progression prior to any chemotherapeutic treatments, indicating the importance of its mutations in the initiation and propagation of AML^20^. Additionally, it has been shown that *p53* mutations are strongly associated with transformation of AML in patients into myeloproliferative neoplasms, suggesting their vital involvement during the leukemic transformations^21^.

In spite of these findings, neither the precise molecular mechanisms behind the transcriptional regulation of *CBFB* nor the functional/physical association between CBFB and p53 has so far remained entirely elusive. Furthermore, the actual molecular basis of how AML cells could adapt to RUNX1-attenuated environment has been largely unknown. Here, we have sought to clarify the transcriptional regulatory mechanisms of *CBFB* and also examined the presence of the cell-autonomous compensation mechanisms after *RUNX1-*inhibition therapy in AML cells.

## Results

### p53 transcriptionally regulates *CBFB* expression

**Figure 1:**
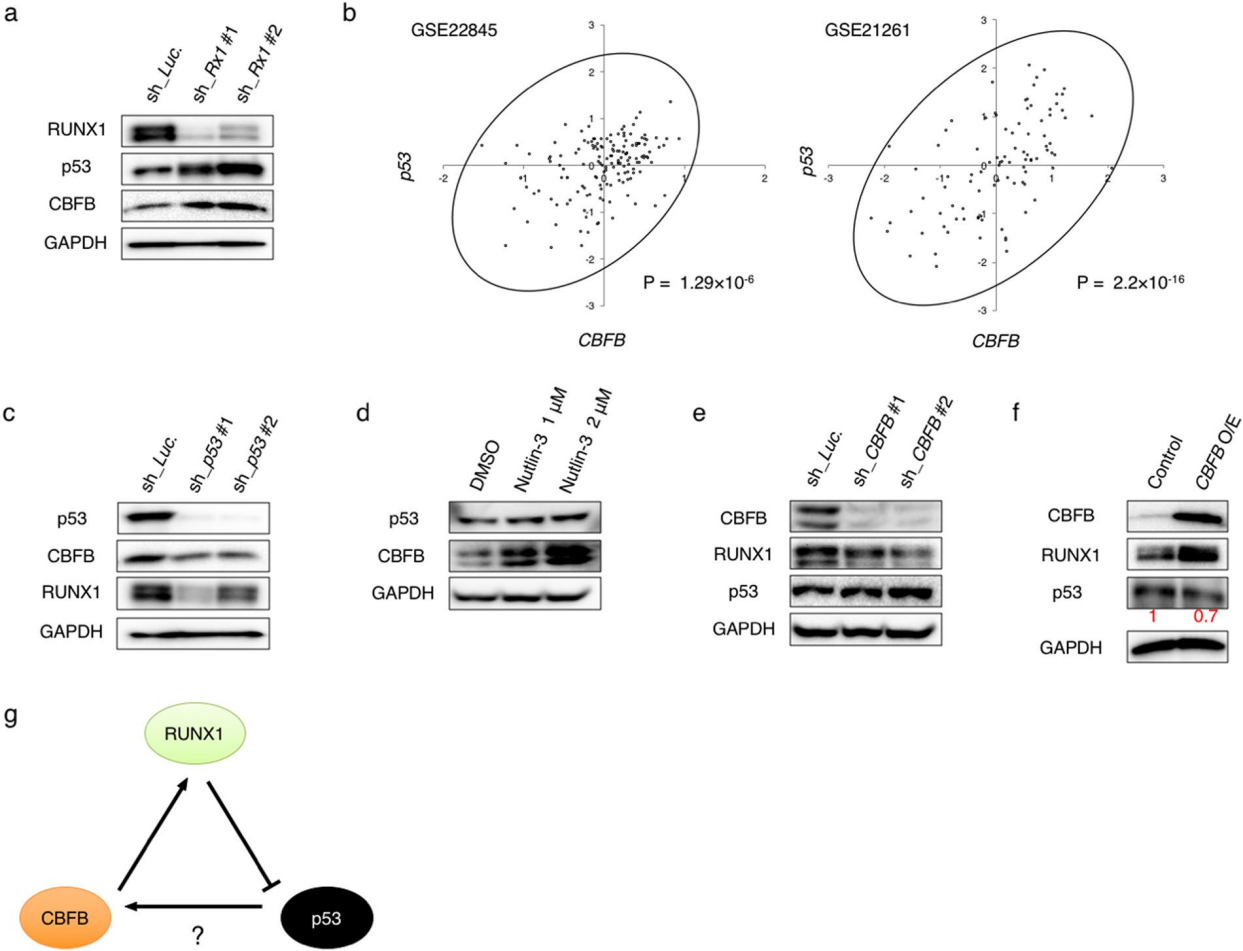
p53 induces CBFB expression in AML cells. (**a**) *RUNX1* depletion induces p53 and CBFB. MV4-11 cells were lentivirally-transduced with control (sh_*Luc*) or with shRNAs targeting *RUNX1* (sh_*Rx1* #1 and sh_*Rx1* #2) and treated with 3 μM doxycycline. Forty-eight hours after treatment, cell lysates were prepared and analyzed by immunoblotting with the indicated antibodies. GAPDH was used as a loading control. (**b**) Correlation between p53 and CBFB expressions in AML patients from 2 independent clinical datasets (GSE22845; n = 154, GSE21261; n = 96). *P* value by Spearman’s correlation. (**c**) Knockdown of *p53* promotes down-regulation of CBFB and RUNX1. MV4-11 cells were lentivirally-transduced with control (sh_*Luc*) or with shRNAs targeting *p53* (sh_*p53* #1 and sh_*p53* #2) and treated as in (**a**). Cell lysates were analyzed by immunoblotting with the indicated antibodies. GAPDH was used as a loading control. (**d**) Nutlin-3 exposure induces CBFB. MV4-11 cells were treated with Nutlin-3 for 24 hours at the indicated concentrations. After the treatment, cell lysates were analyzed by immunoblotting with the indicated antibodies. GAPDH was used as a loading control. (**e**) Depletion of *CBFB* causes down- and up-regulation of RUNX1 and p53, respectively. MV4-11 cells lentivirally-transduced with control (sh_*Luc*.) or shRNAs targeting *CBFB* (sh_*CBFB* #1 and sh_*CBFB* #2) were treated as in (**a**). Forty-eight hours after the treatment, cell lysates were analyzed by immunoblotting with the indicated antibodies. GAPDH was used as a loading control. (**f**) Forced expression of CBFB increases and decreases RUNX1 and p53, respectively. MV4-11 cells were transduced with control lentivirus or with lentivirus encoding CBFB (*CBFB* O/E) and treated as in (**a**). Forty-eight hours after the treatment, cell lysates were analyzed by immunoblotting with the indicated antibodies. GAPDH was used as a loading control. Signal intensities of p53 in each lane were measured by Image Lab software and shown in red. (**g**) Working model of our present study. *RUNX1* depletion induces p53, which results in an increase in CBFB expression level. Intracellular CBFB accumulation stabilizes the residual RUNX1 to compete the RUNX1-inhibition therapy, thus creating the cell-autonomous feedback regulatory loop of RUNX1-p53-CBFB in AML cells.

To investigate *RUNX1* depletion-mediated cellular responses, we have constructed tetracycline-inducible shRNAs targeting *RUNX1* (sh_*RUNX1* #1 and #2) and lentivirally-transduced them into AML-derived MV4-11 cells. As shown in Fig. 1a, *RUNX1* gene silencing significantly induced wild-type p53 expression in MV4-11 cells as described previously^5^. We have also found that, like p53, CBFB expression is increased upon *RUNX1*-knockdown. The total amount of *RUNX* family members (*RUNX1* + *RUNX2* + *RUNX3*), which was estimated by RT-qPCR with primer set specifically amplifying the common coding sequence of all RUNX family members, was decreased in these *RUNX1*-knocked down AML cells (Supplementary Fig. S1a). In addition, the simultaneous knockdown of *RUNX1* plus *RUNX2* and/or *RUNX3* further stimulated CBFB expression as compared to that in the absence of *RUNX1* alone. We also found that these CBFB up-regulations are proportional to the extent of p53 induction in these cells (Supplementary Fig. S1b).

Previously, Berardi MJ, Warren AJ and Yan J *et al*. reported that RUNX family members and CBFB form a heterodimeric complex and this complex formation stabilizes RUNX1/CBFB complex on its target DNA^6,22,23^, raising a possibility that depletion of *RUNX1* might disrupt the stable RUNX1/CBFB complex and then destabilize CBFB. Contrary to this hypothesis, our present results clearly showed that the amount of CBFB is remarkably increased upon *RUNX* knockdown under our experimental conditions, indicating that the above-mentioned previous hypothesis is not the case. Considering that the anti-tumor potency of *RUNX* gene silencing is highly dependent on functional p53 with the sequence-specific transactivation capability, we have sought to examine a possible involvement of p53 in the transcriptional regulation of *CBFB*. Close inspection of the gene expression profiles in *de novo* AML patients from two independent studies revealed the presence of the positive correlation between the expression levels of *p53* and *CBFB* (Fig. 1b). In a good agreement with these observations, knockdown of *p53* in MV4-11 cells caused an obvious reduction in CBFB as well as RUNX1 relative to non-silencing control cells both at the mRNA and protein levels (Fig. 1c, Supplementary Fig. S2a). When MV4-11 cells were exposed to p53 inducer Nutlin-3^24^, CBFB level was up-regulated in a dose-dependent manner (Fig. 1d). In addition, the expression level of RUNX1 was reduced in *CBFB*-knocked down MV4-11 cells, whereas p53 was induced in response to *CBFB* depletion (Fig. 1e). To further confirm these results, CBFB was overexpressed in MV4-11 cells. As expected, forced expression of CBFB resulted in a significant increase and decrease in RUNX1 and p53, respectively (Fig. 1f). These observations raise a possibility that AML cells lacking RUNX1 might survive due to the enhanced expression of CBFB. Given the positive correlation between the expression levels of *p53* and *CBFB* in a variety of AML cases as shown in Fig. 1b, it is likely that *RUNX1* depletion-mediated up-regulation of CBFB is under the control of the accumulated p53, and thus creating an autonomous RUNX1-p53-CBFB feedback loop regulatory system for AML cell proliferation (Fig. 1g).

**Figure 2:**
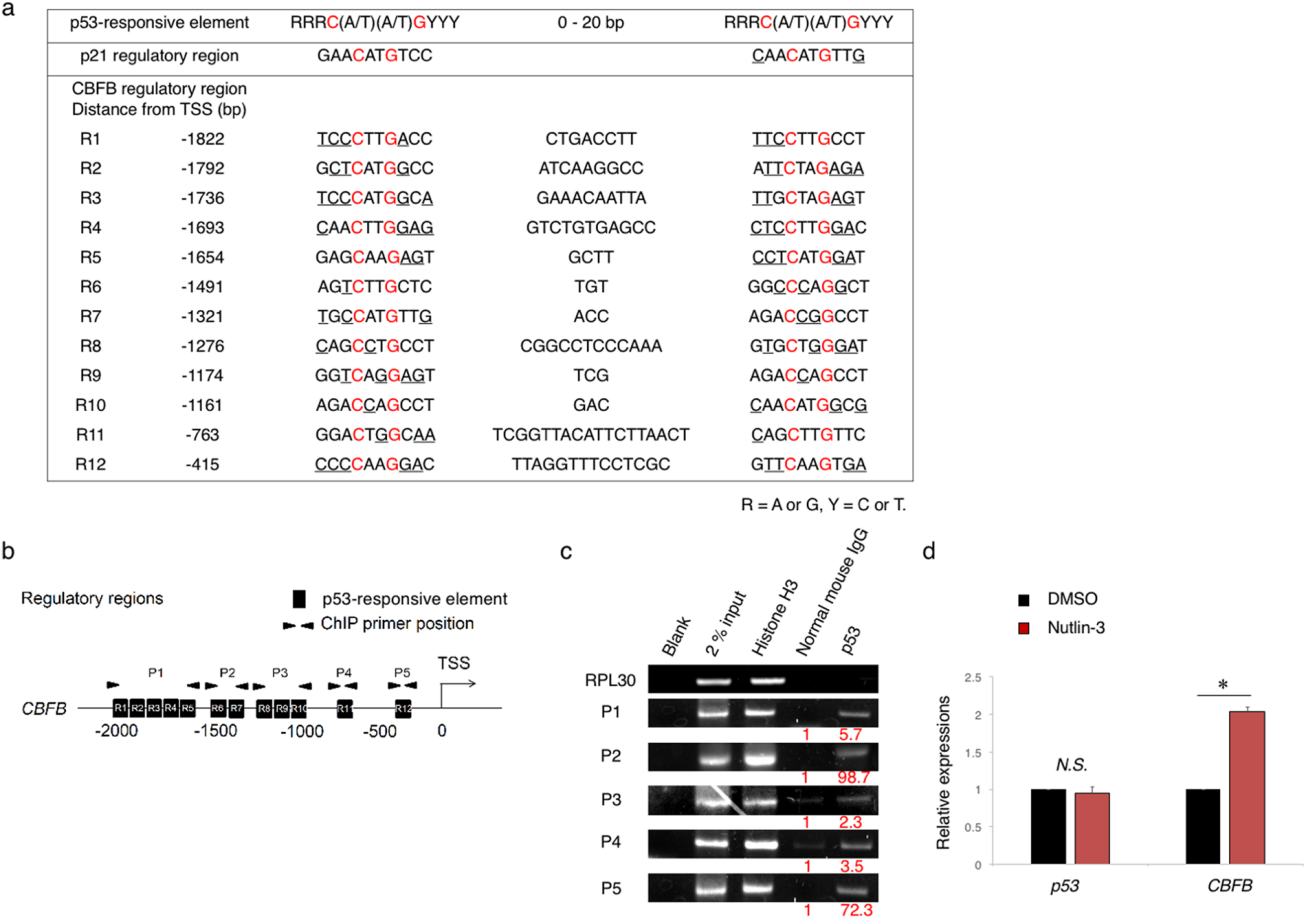
p53 directly transactivates CBFB expressions. (**a**) List of the putative p53-responsive elements within 5′-upstream region of *CBFB*. (**b**) Schematic drawing of 5′-upstream region of *CBFB*. The positions of 12 canonical p53-responsive elements (R1-R12) and the primer sets (P1- P5) used for ChIP analysis are shown. (**c**) p53 binds to *CBFB* promoter region. MV4-11 cells were treated with 1 μM of Nutlin-3. Twenty-four hours after treatment, cells were cross-linked and immunoprecipitated with anti-p53 antibody, an isotope-matched control IgG or with anti-Histone H3 antibody. ChIP products were subjected to PCR-based amplification with the indicated primer sets (see Supplementary Table S2), and RPL30 as a negative control. (**d**) Nutlin-3 treatment induces *CBFB* transcription. MV4-11 cells were exposed to 1 μM of Nutlin-3. Twenty-four hours post treatment, total RNA was prepared and analyzed by real-time RT-PCR. Values are normalized to that of DMSO treated cells (n = 3). Data are mean ± SEM. *P < 0.05, by two-tailed Student’s *t*-test.

Intriguingly, we have found out multiple p53-responsive element-like sequences within the putative *CBFB* promoter region (at positions from −2000 to +200 relative to the transcription start site (+1)) (Fig. 2a and b). Kenzelmann BD and Li M *et al*. have previously demonstrated that p53 bound to *CBFB* promoter region as examined by chromatin immunoprecipitation (ChIP) with anti-p53 antibody followed by DNA sequencing^25,26^. In accordance with these results, the indicated *CBFB* genomic fragments (P1, P2, P3, P4 and P5) containing the possible p53-responsive element-like DNA sequences were detectable in anti-p53 immunoprecipitates under our experimental conditions (Fig. 2c). Additionally, Nutlin-3 treatment markedly stimulated *CBFB* transcription (Fig. 2d). In luciferase reporter assay with *CBFB* promoter region (−1884 bp to +150 bp of transcriptional start site (TSS)), Nutlin-3 treatment indeed up-regulated the *CBFB* promoter activity (Supplementary Fig. S3a). Together, these data strongly suggest the vital role of p53 in the transcriptional regulation of *CBFB*.

### Unidirectional compensatory regulatory loop of RUNX1-p53-CBFB

**Figure 3:**
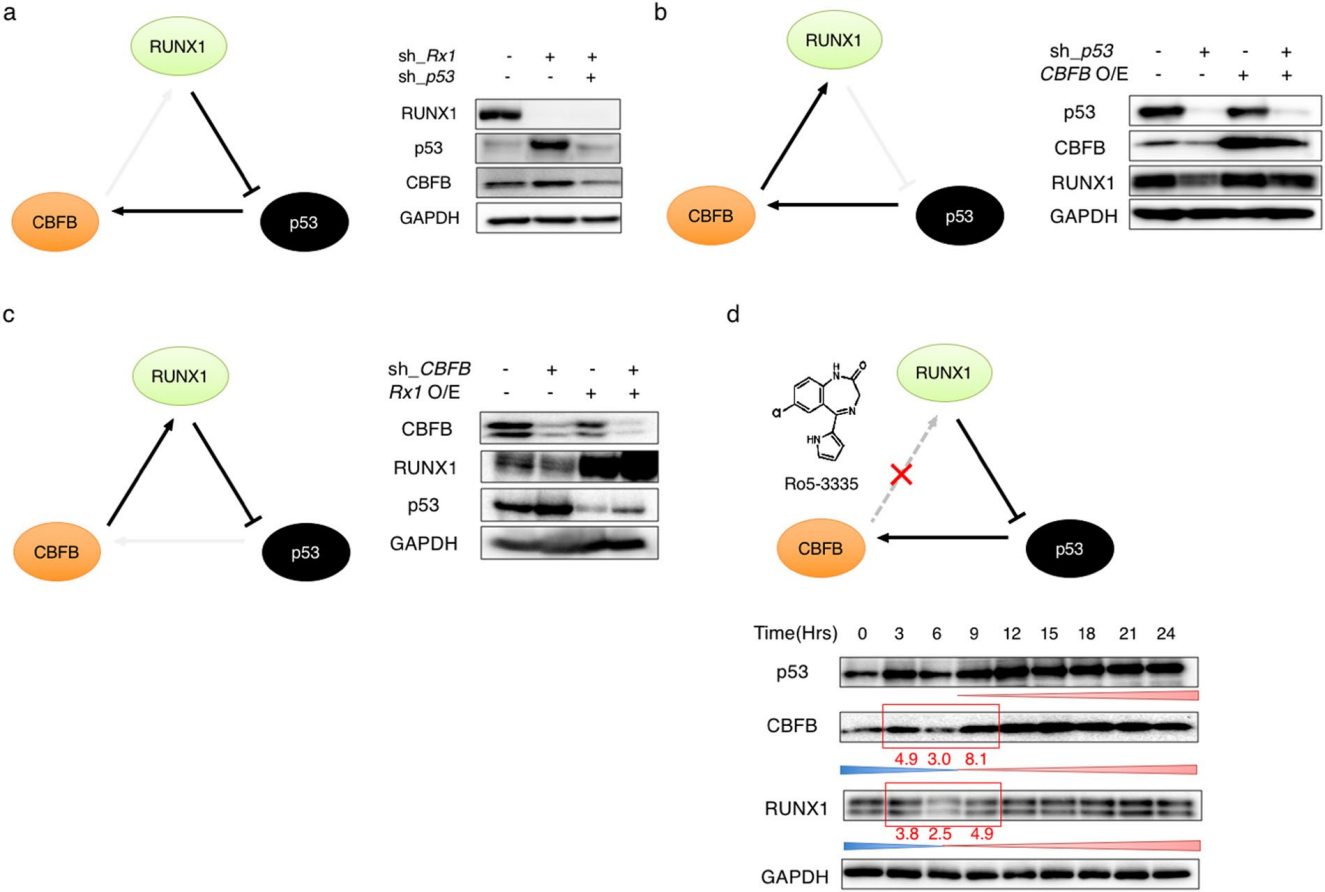
Unidirectional regulatory loop of RUNX1-p53-CBFB. (**a**) *RUNX1*-depletion-mediated up-regulation of CBFB is attenuated by the additional knockdown of *p53* (left panel). MV4-11 cells were lentivirally-transduced with the indicated combinations of shRNAs, and treated with 3 μM of doxycycline. Forty-eight hours after treatment, cell lysates were analyzed by immunoblotting with the indicated antibodies. GAPDH was used as a loading control (right panel). (**b**) *p53*-depletion-mediated decrease in the amount of RUNX1 is restored by ectopic expression of CBFB (left panel). MV4-11 cells were transduced with the indicated combinations of lentivirus vectors and treated with 3 μM of doxycycline. Forty-eight hours after treatment, cell lysates were analyzed by immunoblotting with the indicated antibodies. GAPDH was used as a loading control (right panel). (**c**) *CBFB*-depletion-mediated increase in the amount of p53 is attenuated by forced expression of RUNX1 (left panel). MV4-11 cells were transduced with the indicated combinations of lentivirus vectors and treated with 3 μM of doxycycline. Forty-eight hours after treatment, cell lysates were analyzed by immunoblotting with the indicated antibodies. GAPDH was used as a loading control (right panel). (**d**) The presence of unidirectional regulatory loop of RUNX1-p53-CBFB (upper panel). MV4-11 cells were exposed to 2 μM of Ro5-3335. At the indicated time points after treatment, cell lysates were analyzed by immunoblotting with the indicated antibodies. GAPDH was used as a loading control (lower panel). Signal Intensities of the indicated bands were quantitated by Image Lab software.

To further confirm the existence of RUNX1-p53-CBFB regulatory loop, we have conducted a series of gene knockdown and restore experiments in AML cells. As shown in Fig. 3a, the additional knockdown of *p53* in *RUNX1*-depleted MV4-11 cells reduced the expression level of CBFB to the baseline level. Together with the results shown in Fig. 1a, these data imply that p53 induced by *RUNX1* gene silencing subsequently promotes the expression of CBFB in AML cells. While, knockdown of *p53* led to a significant suppression of CBFB as well as RUNX1, and the decreased expression level of RUNX1 was restored by overexpression of CBFB (Fig. 3b). These observations are suggestive that CBFB induced by p53 subsequently stabilizes RUNX1 in AML cells. Finally, we have examined the expression levels of RUNX1 and p53 in *CBFB*-knocked down MV4-11 cells. As seen in Fig. 3c, *CBFB* gene silencing decreased and increased the expression levels of RUNX1 and p53, respectively. As expected, forced expression of RUNX1 in *CBFB*-depleted MV4-11 cells caused a reduction in p53 to the control level. These results indicate that silencing of *CBFB* destabilizes RUNX1, which in turn induces p53 in AML cells. Consistent with these findings, the down-regulation of RUNX1 was detectable in MV4-11 cells at 6 hours after the treatment with Ro5-3335, which has been shown to facilitate the specific dissociation of RUNX1 from CBFB^27^ (Fig. 3d). Nine hours after Ro5-3335 treatment, the amounts of p53 and CBFB were increased in a time-dependent manner. Collectively, these results strongly suggest the presence of unidirectional compensatory circuit of RUNX1-p53-CBFB in AML cells.

### RUNX1-p53-CBFB axis confers resistance to anti-leukemia therapy

**Figure 4:**
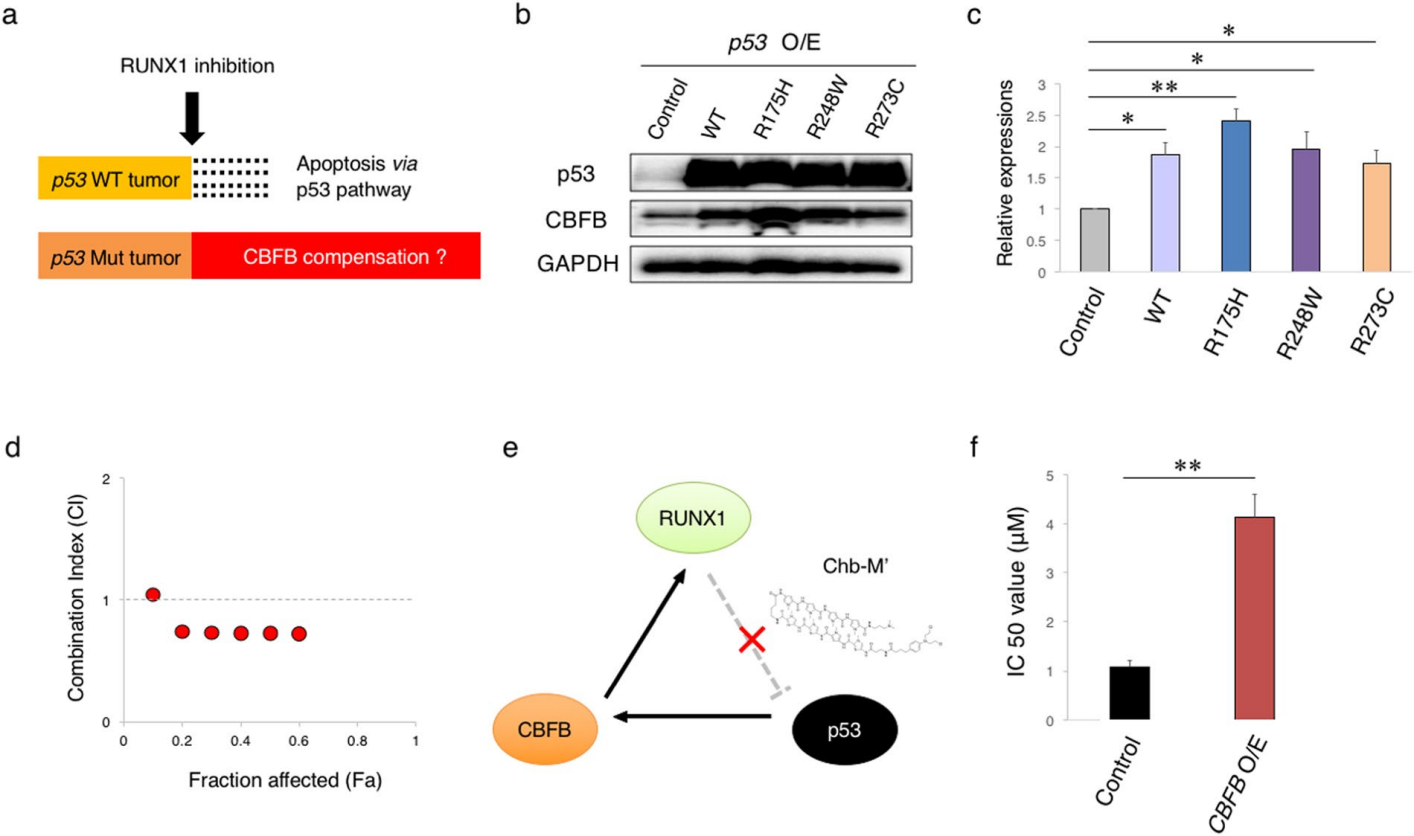
Mutated p53 induces CBFB. (**a**) Schematic abstract showing the different biological response to RUNX1 inhibition of *p53*-proficient and *p53*-mutated tumors. (**b**) p53 mutants induce the expression of CBFB. HEK293T cells were transfected with the indicated expression plasmids. Forty-eight hours after transfection, cell lysates were analyzed by immunoblotting with the indicated antibodies. GAPDH was used as a loading control. (**c**) Cumulative results of (**a**). Signal intensities of the bands for CBFB were measured by Image Lab software and adjusted to those of GAPDH bands. Values were normalized to that of control samples (n = 3). (**d**) Combination index plots of Ro5-3335 and CP-31398 in *p53*-mutated (MV4-11NR) AML cells (n = 3). (**e**) Schematic illustration showing the interaction site of Chb-M’ in the RUNX1-p53-CBFB loop. Chb-M’ binds to RUNX1-consensus binding sequences on the genome DNA, inhibits the recruitment of RUNX1 onto its target sites, and thereby activating p53 pathway. (**f**) IC50 values of Chb-M’ in MV4-11 cells transduced with the empty lentivirus or with the lentivirus for CBFB (CBFB O/E). Cells were simultaneously treated with 3 μM of doxycycline and various concentrations of Chb-M’ for 48 hours (n = 3). Data are mean ± SEM. *P < 0.05, **P < 0.01, by two-tailed Student’s *t*-test.

Although the majority of primary *de novo* AML cases harbor wild-type *p53*, a small number of AML patients do carry *p53* mutations. Since over 90% of *p53* mutations occur within its sequence-specific DNA-binding domain, it is indicative that mutated p53 has an ability to recognize and bind to DNA sequences distinct from those of wild-type p53. On the other hand, it has also been described in several reports that p53-target sequences remain unchanged regardless of *p53* status^8,9,11,13^. According to our RUNX1-p53-CBFB loop working model, the stabilized mutant p53 possibly augments this feedback regulatory loop through the direct transactivation of *CBFB* and potentially contributes to the acquired resistance to RUNX1-inhibition therapy (Fig. 4a). We thus examined whether p53 mutants could up-regulate CBFB expression. To this end, we have generated three expression plasmids of representative p53 mutants (R175H, R248W and R273C) and introduced them into HEK293T cells. As shown in Fig. 4b and c, like wild-type p53, these mutants significantly induced CBFB. In luciferase reporter assays using *CBFB* promoter (Supplementary Fig. 4a), these mutants retained ability enough to transactivate *CBFB* expressions. As we have expected, ChIP assay with anti-p53 antibody in p53-mutated AML cell line MV4-11NR (R248W) revealed the binding of mutant p53 to the CBFB promoter region. In addition, wild-type p53 inducer CP-31398^28^ and RUNX1-CBFB inhibitor Ro5-3335 have synergistically worked (combination index (CI) < 1 at fraction of affected (Fa) = 0.5) in MV4-11NR cells bearing *p53* mutation, which were originally resistant to Ro5-3335 treatment (Fig. 4d). Consistent with these findings, knockdown of mutant p53 in MV4-11NR cells significantly suppressed CBFB expression (Supplementary Fig. 4c). MDM2 inhibition with Nutlin-3 didn’t induce enough p53 elevation in MV4-11NR cells, probably due to the saturated expression of stablized mutant p53 in these cells (Supplementary Fig. 4d). These findings collectively indicated that even a mutated p53 can transactivate *CBFB* expressions.

We have previously reported a potent RUNX inhibitor Chb-M’ and its efficacy in AML cells^5^. Based on our previous results, Chb-M’ impaired the interaction of RUNX family members with their consensus binding sequences on the genome DNA, and then exerted its anti-leukemic effect (Fig. 4e). Consistent with our current findings, MV4-11 cells expressing an exogenous CBFB acquired the resistance to Chb-M’ (Fig. 4f). Therefore, these results indicate that *p53* mutations possibly confer resistance to RUNX1-inhibition therapy through the induction of CBFB.

**Figure 5:**
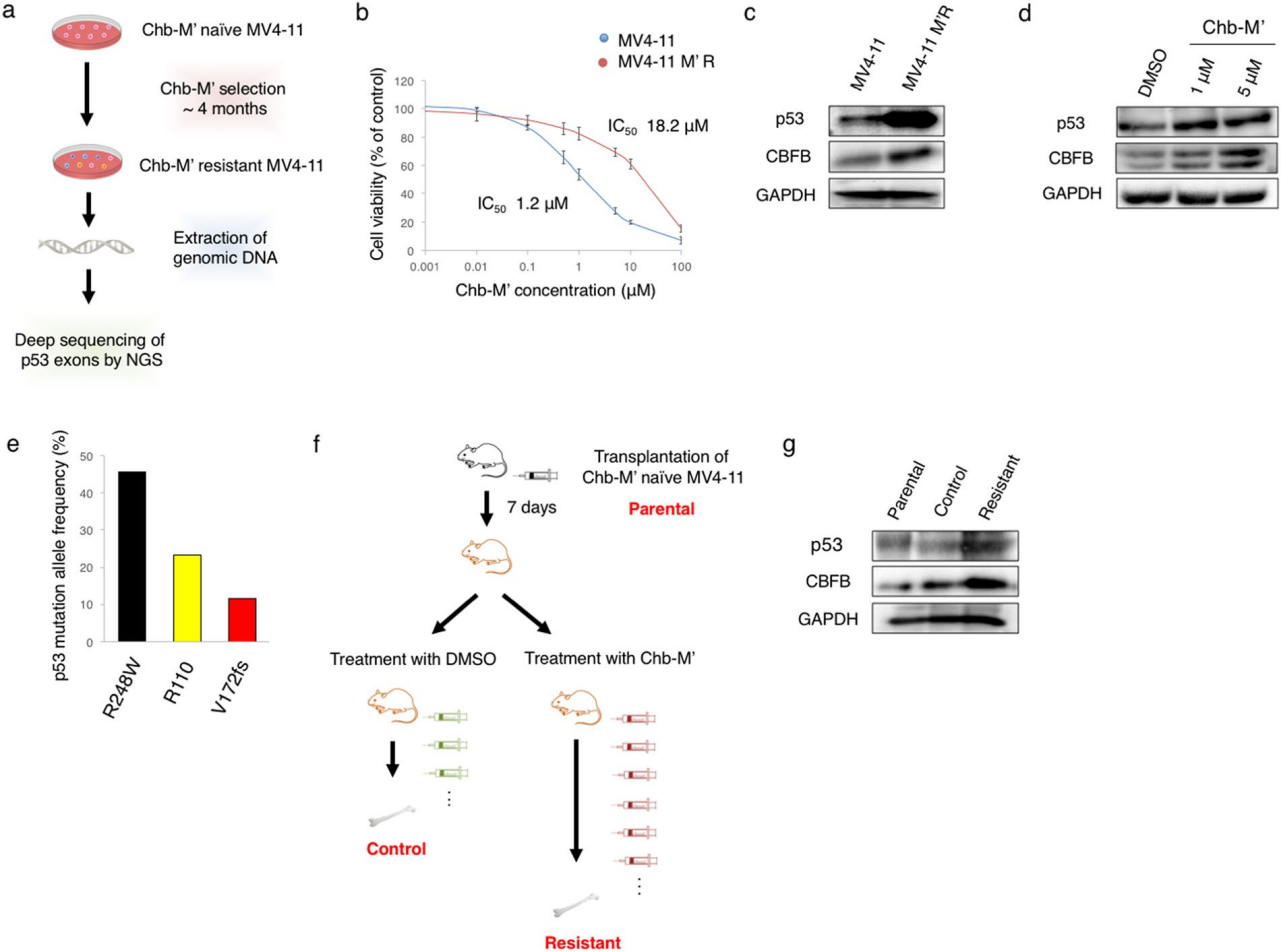
Mutated p53-dependent induction of CBFB contributes to the resistance to RUNX1 inhibition therapy. (**a**) Schematic diagram of the procedure to establish Chb-M’-resistant MV4-11 clones (MV4-11M’R) and subsequent mutation analysis by next generation sequencing (NGS). (**b**) Calculation of IC50 of Chb-M’ in Chb-M’-naïve and Chb-M’-resistant MV4-11 cells (MV4-11M’R) (n = 3). (**c**) Induction of p53 and CBFB in MV4-11 M’R cells. Cell lysates prepared from the parental MV4-11 and MV4-11M’R cells were analyzed by immunoblotting with the indicated antibodies. GAPDH was used as a loading control. (**d**) Chb-M’ treatment further stimulates the expression levels of p53 and CBFB in MV4-11M’R cells. MV4-11M’R cells were exposed to the indicated concentrations of Chb-M’. Twenty-four hours after treatment, cell lysates were prepared and subjected to immunoblotting with the indicated antibodies. GAPDH was used as a loading control. (**e**) Frequent *p53* mutation at codon R248W in MV4-11 M’R cells. Genomic DNA was prepared from MV4-11 M’R cells according to the standard procedure, and analyzed for *p53* mutations by next generation sequencing. (**f**) Schematic drawing of the transplantation assay in NOG mice. Chb-M’ treatment (twice/week) was continued until the recipient mice show the sign of leukemia development. AML cells were then extracted from sacrificed mice with leukemia. (**g**) Chb-M’-resistant AML cells highly express p53 and CBFB. Cell lysates prepared from parental, control and resistant cells were analyzed by immunoblotting with the indicated antibodies. GAPDH was used as a loading control.

**Figure 6:**
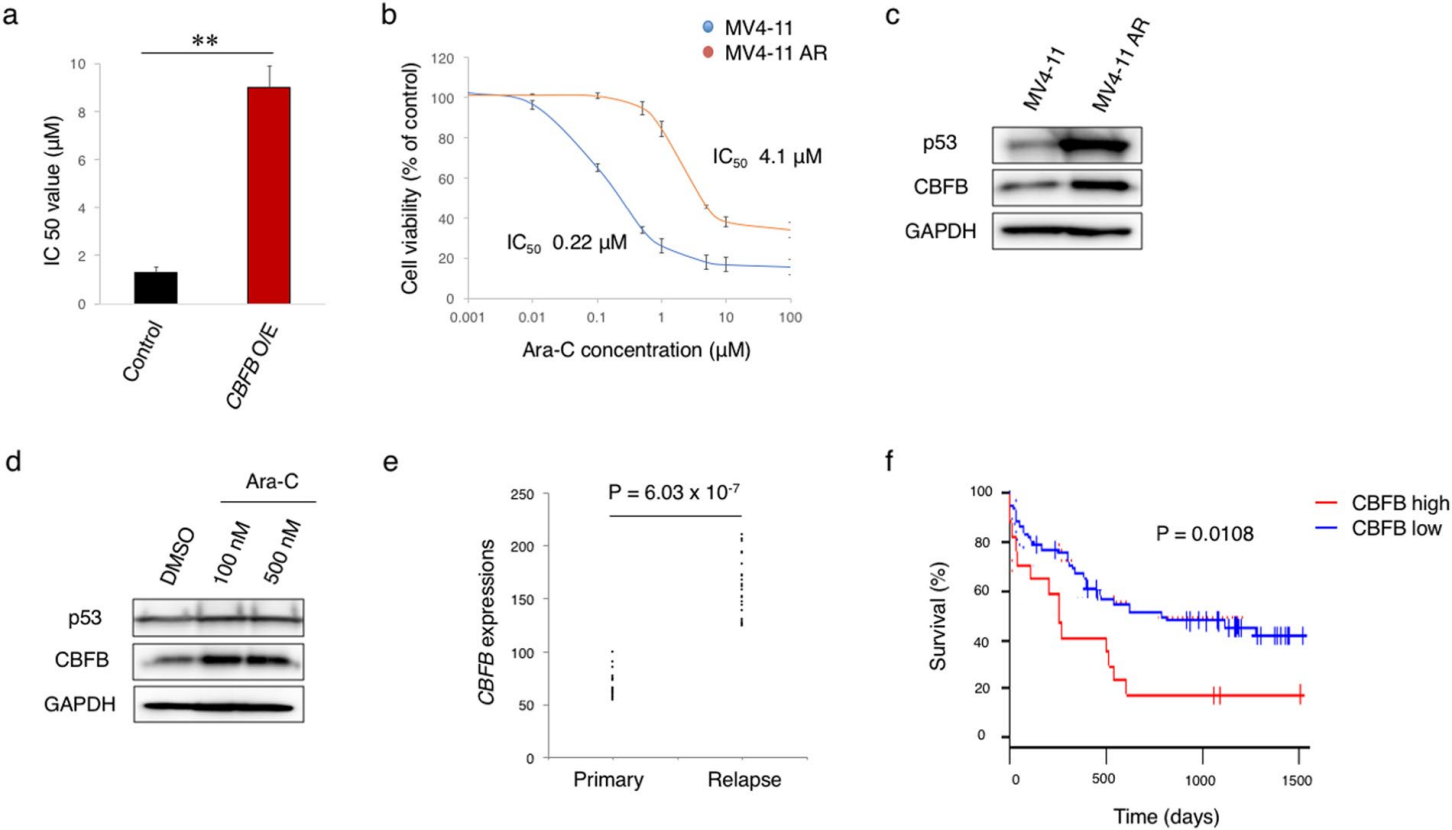
Ectopic expression of CBFB confers resistance to the conventional anti-leukemia therapies. (**a**) IC50 values of Ara-C in CBFB-overexpressed MV4-11 cells. MV4-11 cells were transduced with lentivirus expressing CBFB (CBFB O/E) or control empty lentivirus. Cells were then treated simultaneously with 3 μM doxycycline and various concentrations of Ara-C. Forty-eight hours after treatment, IC50 values were calculated (n = 3). **P < 0.01, by two-tailed Student’s *t*-test. (**b**) IC50 values of Ara-C in Ara-C-naïve MV4-11 cells and Ara-C-resistant MV4-11 cells (MV4-11AR) (n = 3). (**c**) MV4-11AR cells highly express p53 and CBFB. Cell lysates prepared from parental MV4-11 cells and Ara-C-resistant MV4-11AR cells were analyzed by immunoblotting with the indicated antibodies. GAPDH was used as a loading control. (**d**) Ara-C treatment further augments p53 and CBFB expressions in MV4-11AR cells. MV4-11AR cells were treated with DMSO or with the increasing concentrations of Ara-C. Twenty-four hours after treatment, cell lysates were prepared and analyzed by immunoblotting with the indicated antibodies. GAPDH was used as a loading control. (**e**) A higher expression level of CBFB in AML cells obtained from the patients at the relapse phases relative to that at the primary phases (GSE17855 and GSE52891, n = 23). (**f**) Overall survival of AML patients with a higher or with a lower expression level of *CBFB* (GSE12417, high n = 17, low n = 62). *P* value by log-rank (Mantel-Cox) test. Data are mean ± SEM.

To further verify the relationship between *p53* expression/mutation and resistance to RUNX1 inhibition, we have employed the unbiased methods. Firstly, we have cultured Chb-M’-naïve MV4-11 cells in the medium containing a gradually increasing amounts of Chb-M’, and finally established the Chb-M’-resistant MV4-11 clones (MV4-11M’R) through the continuous selection of the resistant cells *in vitro* for up to 4 months (Fig. 5a and b). Intriguingly, the expression analyses demonstrated that MV4-11M’R cells expressed a larger amount of p53 and CBFB than the parental MV4-11 cells (Fig. 5c), and their expression levels have increased in response to Chb-M’ in a dose-dependent manner (Fig. 5d). We then comprehensively examined the mutation frequency of *p53* in MV4-11M’R cells by using the next generation sequencing (NGS) and demonstrated that the mutation frequency of *p53* at codon R248W is around 45% (Fig. 5e), implying that one of 6 hot-spot mutations of *p53* (R248W) occurs in most of the Chb-M’-resistant MV4-11M’R cells. This mutated p53 (R248W) has indeed an ability to up-regulate CBFB expression as shown in Fig. 4b and c. We have also carried out the *in vivo* selection for Chb-M’-resistant clones (Fig. 5f) and found a marked induction of p53 and CBFB in Chb-M’-resistant AML cells (Fig. 5g). These observations strongly suggest that the RUNX inhibition-mediated treatment permits the selective proliferation of *p53*-mutated AML cells and these cells acquire the enhanced tumorigenicity through the potentiated p53-CBFB-RUNX feedback loop. Notably, CBFB-overexpression in MV4-11 cells conferred proliferative advantage and resistance to Ara-C (cytarabine), a widely-used first line clinical anti-leukemia drug (Fig. 6a, Supplementary Fig. 5a). Making a sharp contrast to *CBFB* overexpression, knockdown of *CBFB* in MV4-11 cells conferred significant sensitivity to Ara-C (Supplementary Fig. 5b and c). When all RUNX family expressions were knocked down in *CBFB*-overexpressed MV4-11 cells, CBFB-mediated Ara-C resistance was significantly reverted (Supplementary Fig. 5d and e), suggesting that CBFB-induced drug-resistance was probably mediated by stabilized RUNX family members.

We have also observed that Ara-C-resistant MV4-11 cells (MV4-11AR) express a significant larger amount of CBFB than Ara-C-naïve parental MV4-11 cells (Fig. 6b and c). In addition, Ara-C exposure further augmented CBFB expression in MV4-11AR cells (Fig. 6d). In a good agreement with these results, a larger amount of *CBFB* was detectable in patients’ AML samples obtained at their relapse phase than those obtained at the primary phase (Fig. 6e). Extensive analysis of the clinical datasets^29^ revealed that the overexpression of CBFB confers an accelerated disease progression and shortened overall survival periods not only to AML patients but also to various types of cancers (Fig. 6f and Supplementary Fig. S6). Taken together, our present results strongly suggest that the p53-CBFB-RUNX feedback loop is tightly engaged in the tumorigenesis as well as the acquisition of the serious resistance to chemotherapy including the RUNX-specific inhibition therapy.

## Discussion

The context-dependent oncogenic and oncosuppressive functions of RUNX family members (RUNX1, RUNX2 and RUNX3) have been well-described in multiple studies^1^. A growing body of recent evidence supports the notion that RUNX family is tightly linked to the development and maintenance of AML as well as various types of cancers^3,5,30,31^. On the other hand, the functional significance of their heterodimeric partner, CBFB, in oncogenesis has relatively little been known so far. Recently, we have found the redundant functions of RUNX1, RUNX2 and RUNX3 in tumorigenesis and demonstrated that shRNA-mediated knockdown of *RUNX1* in AML cells reciprocally up-regulates RUNX2 and RUNX3 expressions^5^. From these experiments, we have also found *RUNX1* depletion-mediated induction of CBFB. Moreover, the additional knockdown of *RUNX* family genes augmented *RUNX1* silencing-induced expression of CBFB and p53, which prompted us to investigate the possible interactions of CBFB with p53. Previously, it has been shown that CBFB stabilizes the RUNX transcription complex and enhances its DNA-binding capability^6,22,23^. Since the results obtained from our series of gene knockdown and overexpression experiments did not support the above-mentioned hypothesis, it is likely that there exists an alternative molecular mechanism behind the regulation of CBFB expression in AML cells. Although we have found for the first time that *CBFB* transcription is modulated by RUNX family members, we have failed to find out any consensus RUNX-binding sequences (5′-TGTGGT-3′ or 5′-TGCGGT-3′) within the proximal promoter region of *CBFB*. Besides, previously-reported ChIP-sequencing (ChIP-seq) assays with anti-RUNX1 antibody showed no significant peaks in the regulatory region of *CBFB* (GSE22178 and GSE31221)^5,32,33^. Therefore, it is unlikely that *CBFB* is directly transactivated by RUNX family members. On the other hand, ChIP-seq assays with anti-p53 antibody repeatedly proved p53 bindings to CBFB regulatory region (GSE46240 and GSE26361), which results are consistent with our findings as we have mentioned in the Results section^25,26^. Together with the present results showing that p53 directly transactivates *CBFB* promoter through its p53-responsive element-like sequences, it is conceivable that RUNX1 regulates the expression of *CBFB* not directly but indirectly *via* p53.

**Figure 7:**
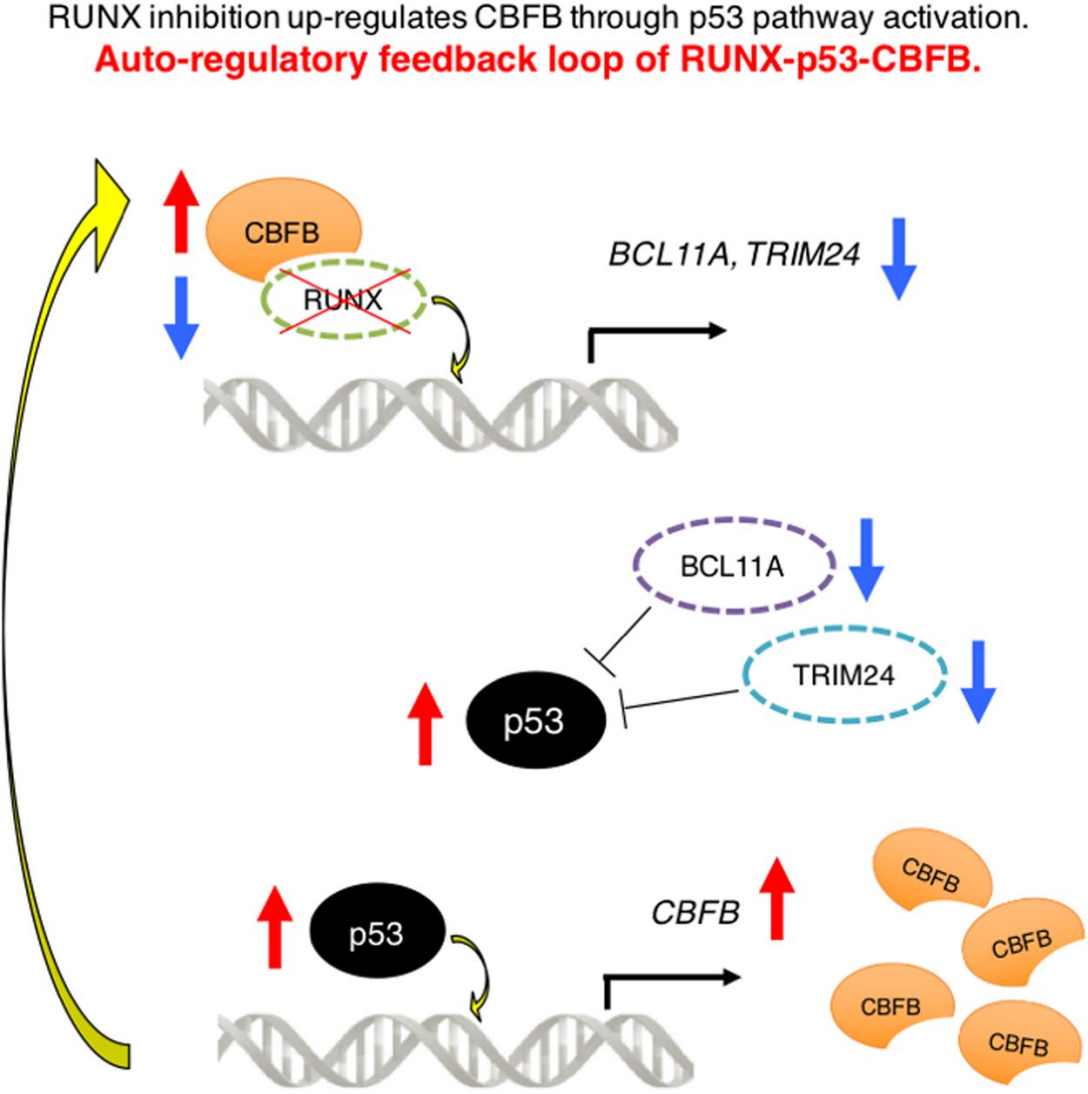
Auto-regulatory feedback loop of RUNX1-p53-CBFB. RUNX1-inhibition treatment induces p53. Induced p53 directly binds to *CBFB* promoter and stimulates its transcription and translation, which in turn acts as a platform for the stabilization of RUNX1, thereby creating the compensative RUNX1-p53-CBFB feedback loop.

In addition to the transcriptional regulatory mechanism of *CBFB*, we have also demonstrated that RUNX1-p53-CBFB regulatory circuit contributes to the acquisition of treatment resistance of AML cells. Collectively, our present study identified the novel molecular mechanism behind *CBFB* regulation and its indispensable roles in tumorigenesis, which provides an insight into understanding how AML cells could become resistant to RUNX1-inhibition therapy (Fig. 7).

In conclusion, our present study strongly suggests that an autonomous RUNX1-p53-CBFB regulatory triangle plays a vital role in the maintenance and the acquisition of chemo-resistance of AML cells, and potentially provides novel therapeutic targets for anti-leukemia strategy.

## Materials and Methods

### Cell lines

AML-derived MOLM13 and MV4-11cells were purchased from Deutsche Sammlung von Mikroorganismen und Zellkulturen GmbH (DSMZ), Germany and American Type Culture Collection (ATCC), USA, respectively. AML-derived MV4-11NR cells harboring *TP53* R248W mutation were kind gift from Dr. T. Ikezoe (Department of Hematology and Respiratory Medicine, Kochi University, Kochi, Japan). Embryonic kidney HEK293T cells were provided from Japanese Collection of Research Bioresources (JCRB), Japan. HEK293T cells were maintained in Dulbecco’s modified Eagle’s medium (DMEM) supplemented with 10% heat-inactivated fetal bovine serum (FBS) and 1% Penicillin-Streptomycin (PS) in incubators with humidified atmospheres of 5% CO_2_ and 95% air at 37 °C. The other cells were cultured in Roswell Park Memorial Institute (RPMI) 1640 medium containing 10% FBS and 1% PS under 5% CO_2_ and 95% air at 37 °C.

### IC50 evaluation

For cell survival assay, cells were seeded at a density of 1 × 10^5^ cells/mL. The indicated concentrations of PI polyamides or drugs were added to the culture medium and cells were incubated for 48 hours. Cell viability was then assessed by WST assay using Cell Count Reagent SF (nacalai tesque, Inc.) and Infinite^®^ 200 PRO multimode reader (TECAN) according to the manufacturer’s instructions. Percent inhibition curves were drawn and IC50 of the indicated compounds was calculated based on the median-effect method^34^.

### Real-time quantitative PCR (qRT-PCR)

Total RNA was isolated by using RNeasy mini kit (Qiagen) and reverse-transcribed with Reverse script kit (TOYOBO) to generate cDNA. Real-time quantitative polymerase chain reaction (PCR) was carried out with 7500 Real-Time PCR System (Applied Biosystems) according to the manufacturer’s recommendations. The results were normalized to *GAPDH* levels. Relative expression levels were calculated using the 2-ΔΔCt method. Primers used for qRT-PCR were listed in Supplementary Table S1.

### ChIP-qPCR

Chromatin immunoprecipitation assay (ChIP) was performed using SimpleChIP® Plus Enzymatic Chromatin IP Kit (Cell Signaling Technology, USA) according to the manufacturer’s protocols. In brief, cells were cross-linked in 1% formaldehyde in PBS for 10 min at room temperature. After glycine quenching, cell pellets were collected, lysed and then subjected to sonication with Q55 sonicator system (QSONICA, USA). The supernatant was diluted with the same sonication buffer and processed for immunoprecipitation with the following antibodies at 4 °C overnight. Anti-p53 antibody (1C12, #2524, Cell Signaling Technology). The agarose beads were washed, chromatin DNA was reverse cross-linked and purified by ethanol precipitation. Following ChIP, precipitated DNA was quantified by qPCR using the standard procedures for 7500 Real-Time PCR System (Applied Biosystems). Primers used for ChIP-qPCR were listed in Supplementary Table S2.

### siRNA interference

Specific shRNAs targeting human *RUNX1*, *RUNX2*, *RUNX3*, *CBFB* and *TP53* were designed and sub-cloned into pENTR4-H1tetOx1, CS-RfA-ETBsd, CS-RfA-ETV and CS-RfA-ETR vectors (RIKEN BRC). Non-targeting control shRNA was designed against *luciferase* (sh_*Luc*). The target sequences were provided in Supplementary Table S3.

### Expression plasmids

Human *RUNX1*, *CBFB* and *p53* cDNAs were amplified by PCR and then inserted into CSII-EF-MCS-IRES2-Venus, CSII-EF-MCS-IRES2-hKO1 and CSIV-TRE-Ubc-KT expression vectors. Series of p53 point mutations (R248W, R175H and R273C) were created by KOD -Plus- Mutagenesis Kit (TOYOBO Co, Ltd.). All of the PCR products were verified by DNA sequencing.

### Production and transduction of lentivirus

For the production of lentivirus, HEK293T cells were transiently co-transfected with lentivirus vectors such as psPAX2 and pMD2.G by polyethylenimine (PEI, Sigma-Aldrich). Forty-eight hours after transfection, viral supernatants were collected and immediately used for infection, and then successfully transduced cells were sorted by flow cytometer Aria III (BD Biosciences) based on the immunofluorescence (Kusabira-Orange or Venus).

### Immunoblotting

Immunoblotting was conducted as described previously^35^. Membranes were probed with the following primary antibodies: anti-RUNX1 (A-2, Santa Cruz Biotechnology, Inc.), anti-GAPDH (FL-335, Santa Cruz Biotechnology, Inc.), anti-RUNX2 (D1L7F, Cell Signaling Technology), anti-RUNX3 (D6E2, Cell Signaling Technology), anti-CBFB (FL-182, Santa Cruz Biotechnology, Inc.) and anti-p53 (1C12, Cell Signaling Technology) antibodies. HRP-conjugated anti-rabbit IgG and anti-mosue IgG (Cell Signaling Technology) were used as the secondary antibodies. Blots were visualized using Chemi-Lumi One Super (nacalai tesque, Inc.) and ChemiDoc^TM^ XRS + Imager (Bio-Rad Laboratories, Inc.) according to the manufacturers’ recommendations. Protein levels were quantified with Image Lab Software (Bio-Rad Laboratories, Inc.).

### Next generation sequencing

Deep sequencing of target exons of *TP53* was performed as described previously with slight modifications^36^. Briefly, tumor DNA specimens prepared from Chb-M’-naïve and -resistant MV4-11 cells were analyzed for possible mutations in TP53. After the extraction of genomic DNA using DNeasy Blood & Tissue Kit (QIAGEN), its concentration was measured using the PicoGreen® reagents (Thermo Fisher Scientific) according to the manufacturer’s instructions. Next, the entire coding sequences of TP53 gene were amplified by independent genomic PCR with a NotI linker attached primer (See Supplementary Table S4 for specific PCR primers). After checking the successful amplification by agarose gel electrophoresis, the PCR products from each sample were combined together, followed by purification of DNA using FastGene Gel/PCR Extraction Kit (Nippon Genetics) and digestion with NotI. The digested DNA was purified again and an aliquot of 2.5 μg of purified DNA was ligated with T4 DNA ligase for 5 hours, sonicated into ~200 bp in length on average using Covaris®, and used for generation of sequencing libraries using NEBNext Ultra DNA Library Prep Kit for Illumina (New England Biolabs). The libraries were then subjected to deep sequencing on Illumina Miseq® following the standard protocol.

### Luciferase reporter assay

Putative promoter region of CBFB (−1884 bp to +150 bp of TSS) was cloned from the genomic DNA of MV4-11 cells using the following primers; F 5′-CCTTGAGGCTGACAATGAGAG -3′ and R 5′-CCGCTTCCCTTTGTTTCAG -3′, and then subcloned into pGL4.20 [luc2/Puro] vector (Promega). Both pGL4.20 CBFB promoter vector and pRL-CMV control vector (TOYO B-Net Co., LTD.) were co-transfected into HEK293T cells. Promoter activities were measured using PicaGene Dual Sea Pansy Luminescence Kit (TOYO B-Net Co., LTD.) and detected by ARVO × 5 (Perkin Elmer) according to the manufacturer’s instructions.

### Statistics

Statistical significance of differences between control and experimental groups was assessed by a 2-tailed unpaired Student’s *t* test, and declared if the *p* value was less than 0.05. Equality of variances in two populations was calculated with F-test. The results were represented as the average ±SEM values obtained from three independent experiments. In transplantation experiments, animals were randomly allocated to each experimental group and the treatments were given with blinding. To examine the overall survival of cancer patients, PrognoScan software was utilized for data extraction and calculation of minimal *p* value^29^. Survival between the indicated groups was compared using the log-rank test. For the measurement of correlation between mRNA or protein expressions, Spearman’s rank correlation coefficient was used.

### Mice

NOD/Shi-scid, IL-2RγKO (NOG) mice were purchased from Central Institute for Experimental Animals, Japan. Littermates were used as controls in all experiments.

### Xenograft mice model

Xenograft mice models of human cancer cell lines were developed using NOG mice. For leukemia mice models, 2.5 × 10^6^ cells/body of MV4-11 cells were intravenously injected. Peripheral blood (PB) was then collected every week and chimerism was checked by flow cytometer using anti-human CD45 antibody (BD Biosciences). One week after injection, mice were treated with PI polyamides (320 μg/kg body weight, twice a week IV injections) or with the equivalent amount of dimethyl sulfoxide (DMSO).

### Synthesis of PI polyamides

Synthesis of Chb-M’ was conducted as previously reported^5^. Briefly, Py-Im polyamide supported by oxime resin was prepared in a stepwise reaction by Fmoc solid-phase protocol. The product with oxime resin was cleaved with *N*,*N*-dimethyl-1,3-propane diamine (1.0 mL) at 45 °C for 3 h. The residue was dissolved in the minimum amount of dichloromethane and washed with diethyl ether to yield a 59.6 mg. To the crude compound (59.6 mg, 48.1 μmol), a solution of chlorambucil (32.6 mg, 107 μmol), PyBOP (benzotriazole-1-yl-oxy-tris-pyrrolidino-phosphonium hexafluorophosphate) (101 mg, 195 μmol), and *N*,*N*-diisopropylethylamine (100 μL, 581 μmol) in *N*,*N*-dimethylformamide (DMF) (300 μL) was added. The reaction mixture was incubated for 1.5 h at room temperature, washed with diethyl ether and DMF for three times, and dried *in vacuo*. The crude product was purified by reversed-phase flash column chromatography (water with 0.1% trifluoroacetic acid/MeCN). After lyophilization, product was obtained (30.2 mg, 19.8 μmol). Machine-assisted polyamide syntheses were performed on a PSSM-8 (Shimadzu) system with computer-assisted operation. Flash column purifications were performed by a CombiFlash Rf (Teledyne Isco, Inc.) with C18 RediSep Rf Flash Column. Electrospray ionization time-of-flight mass spectrometry (ESI-TOF MS) was performed on a Bio-TOF II (Bruker Daltonics) mass spectrometer by using positive ionization mode and proton nuclear magnetic resonance (^1^H NMR) spectra were recorded with a JEOL JNM ECA-600 spectrometer operating at 600 MHz and in parts per million (ppm) downfield relative to tetramethylsilane used as an internal standard to verify the quality of synthesized PI polyamides.

### Study approval

All animal studies were properly conducted in accordance with the Regulation on Animal Experimentation at Kyoto University based on International Guiding Principles for Biomedical Research Involving Animals. All procedures employed in this study were approved by Kyoto University Animal Experimentation Committee (Permit Number: Med Kyo 14332).

## Electronic supplementary material

Supplementary Data
