# Supplementary material for: Autonomous feedback loop of RUNX1-p53-CBFB in acute myeloid leukemia cells
Source: Sci Rep. 2017 Nov 30;7:16604. doi: 10.1038/s41598-017-16799-z (PMC5709397; doi:10.1038/s41598-017-16799-z)
Supplement: Supplementary file 1 — Supplementary Data [file 41598_2017_16799_MOESM1_ESM.doc]

**Data Supplements**

**Autonomous feedback loop of RUNX1-p53-CBFB in acute myeloid leukemia cells**

**Authors and affiliations:**

Ken Morita^1,*^, Mina Noura^1,*^, Chieko Tokushige^1^, Shintaro Maeda^1^, Hiroki Kiyose^1^, Gengo Kashiwazaki^2^, Junichi Taniguchi^2^, Toshikazu Bando^2^, Kenichi Yoshida^3^, Toshifumi Ozaki^4^, Hidemasa Matsuo^1^, Seishi Ogawa^3^, Paul P. Liu^5^, Tatsutoshi Nakahata^6^, Hiroshi Sugiyama^2^, Souichi Adachi^1,7^ and Yasuhiko Kamikubo^1^.

Supplementary Figures p. 2

Supplementary Tables p. 9

**Supplementary Figures**

**
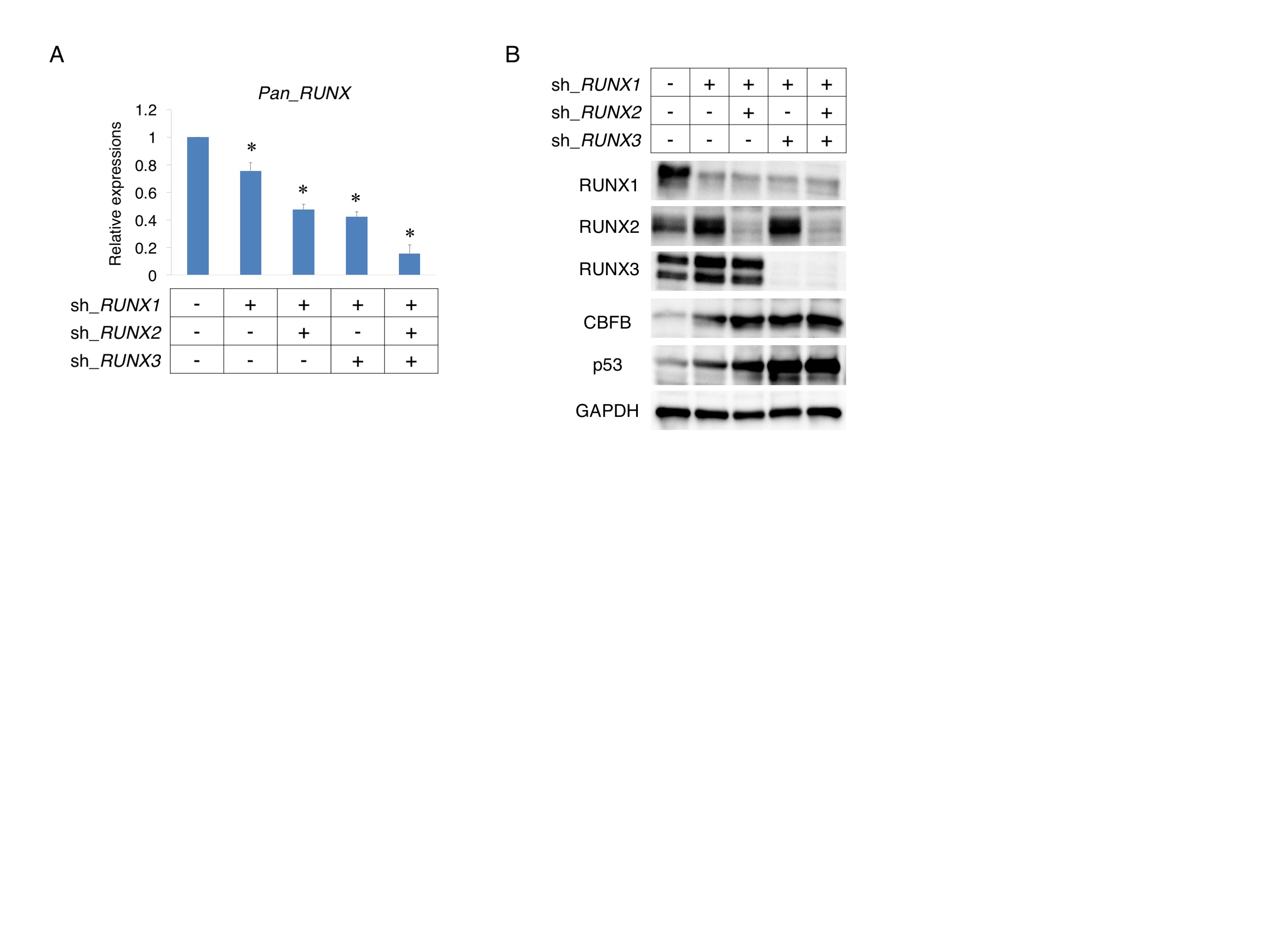
**

**Supplementary Fig. S1**

(A) *Pan_RUNX* expressions were determined in MV4-11 cells transduced with lentivirus encoding shRNA targeting *Luciferase* (sh_*Luc.*) or shRNAs against *RUNX1*, *RUNX2* and *RUNX3*. Cells were incubated with 3μM doxycycline for 48 hours, then total RNA was prepared and analyzed by real-time RT-PCR. Values are normalized to that of control vector-transduced cells (n = 3).

(B) Immunoblot of RUNX1, RUNX2, RUNX3, CBFB, p53 and GAPDH in MV4-11 cells as in (A).

Data are mean±SEM values. * P < 0.05, by two-tailed Student’s *t* test.

**
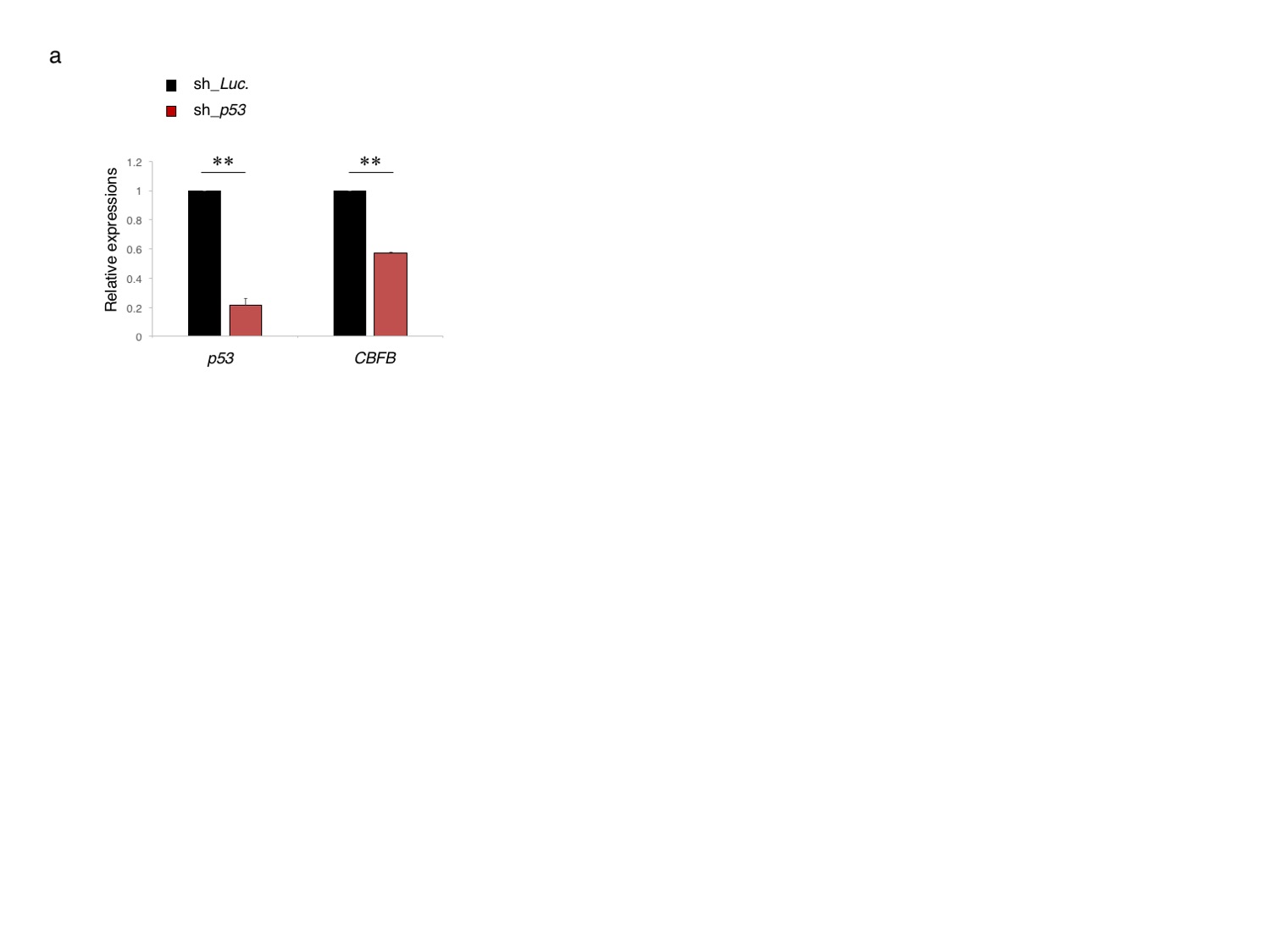
**

**Supplementary Fig. S2**

(a) p53 depletion induces down-regulation of CBFB expression. MV4-11 cells were lentivirally-transduced with shRNAs targeting p53 (sh_*p53*) or control luciferase (sh_*Luc*.), and treated with 3 μM of doxycycline. Twenty-four hours post treatment, total RNA was prepared and analyzed by real-time RT-PCR. Values are normalized to that of control cells (n = 3).

Data are mean ± SEM. ** P < 0.01, by two-tailed Student’s t-test.

**
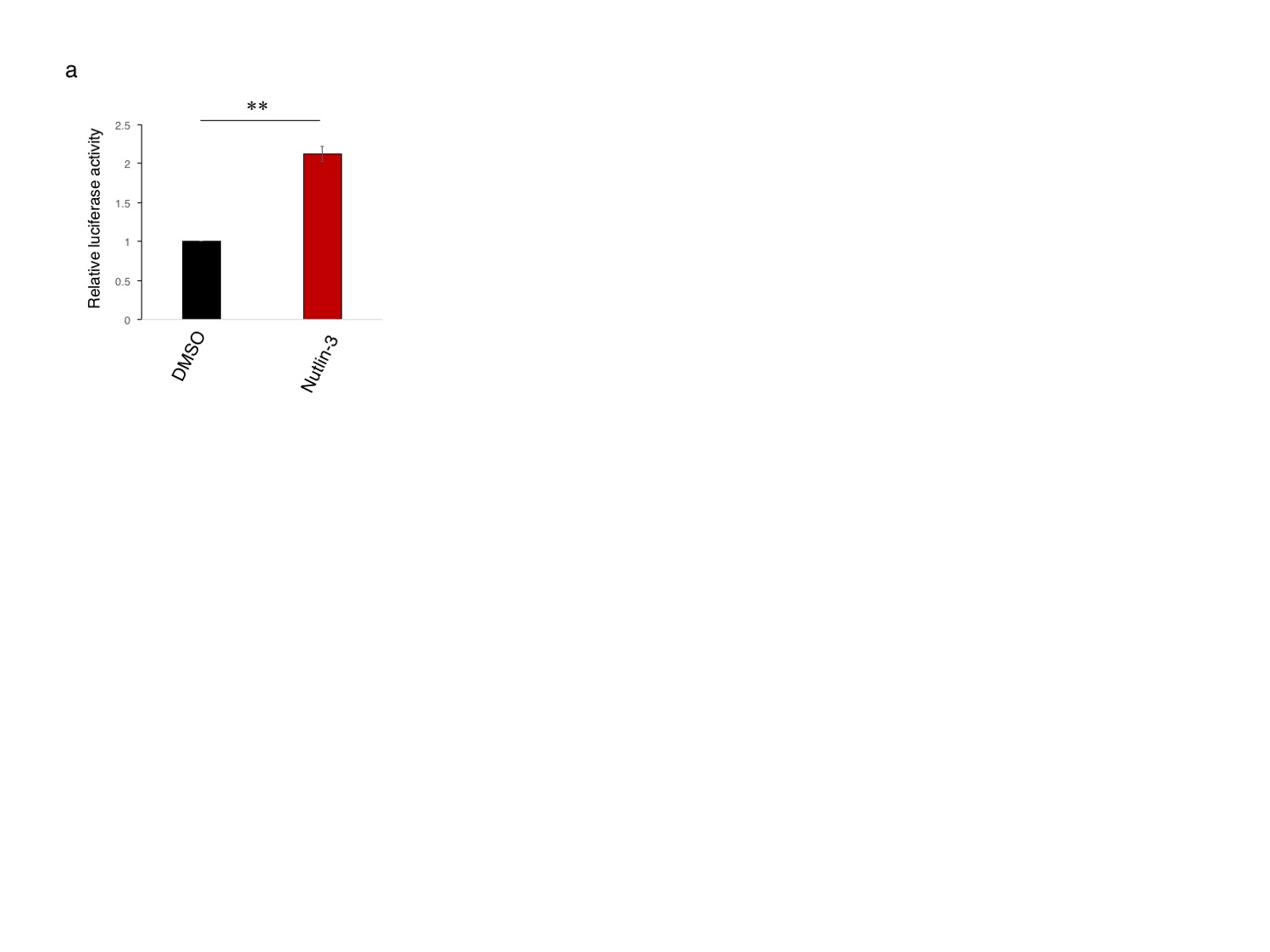
**

**Supplementary Fig. S3**

Luciferase reporter assay of CBFB promoter. HEK293T cells were transfected with luciferase reporter plasmids, then treated with Nutlin-3 at 1 μM or control DMSO. Twenty-four hours after treatment, relative luciferase activity was determined (n = 3).

Data are mean ± SEM. ** P < 0.01, by two-tailed Student’s t-test.

**
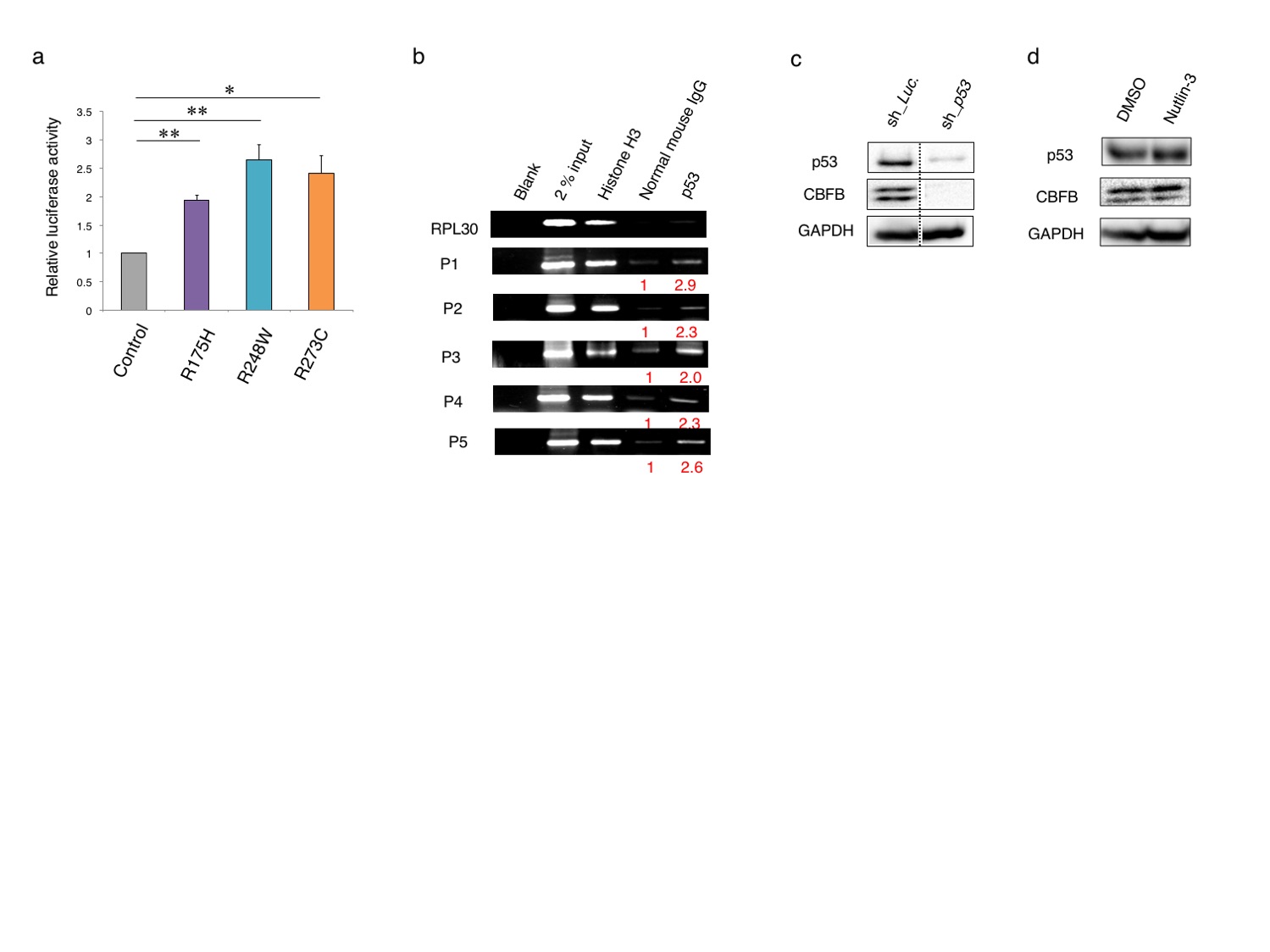
**

**Supplementary Fig. S4**

(a) Luciferase reporter assay of CBFB promoter with mutant p53. HEK293T cells were transfected with luciferase reporter plasmids and mutant p53 expression vectors (R175H, R248W and R273C). Twenty-four hours after transfection, relative luciferase activity was determined (n = 3).

(b) Mutant p53 binds to *CBFB* promoter region. MV4-11NR cells (R248W) were treated with 1 μM of Nutlin-3. Twenty-four hours after treatment, cells were cross-linked and immunoprecipitated with anti-p53 antibody, an isotope-matched control IgG or with anti-Histone H3 antibody. ChIP products were subjected to PCR-based amplification with the indicated primer sets (see Supplementary Table S2), and RPL30 as a negative control.

(c) CBFB expression is down-regulated upon mutant p53-silencing. MV4-11NR cells lentivirally-transduced with control (sh_*Luc*.) or shRNAs targeting *p53* (sh_*p53*) were treated with 3 μM doxycycline. Forty-eight hours after the treatment, cell lysates were analyzed by immunoblotting with the indicated antibodies. GAPDH was used as a loading control.

(d) Nutlin-3 exposure does not induce enough p53 in MV4-11NR cells. MV4-11NR cells were treated with 1 μM of Nutlin-3 for 24 hours. After the treatment, cell lysates were analyzed by immunoblotting with the indicated antibodies. GAPDH was used as a loading control.

Data are mean ± SEM. ** P < 0.01, by two-tailed Student’s t-test.

**
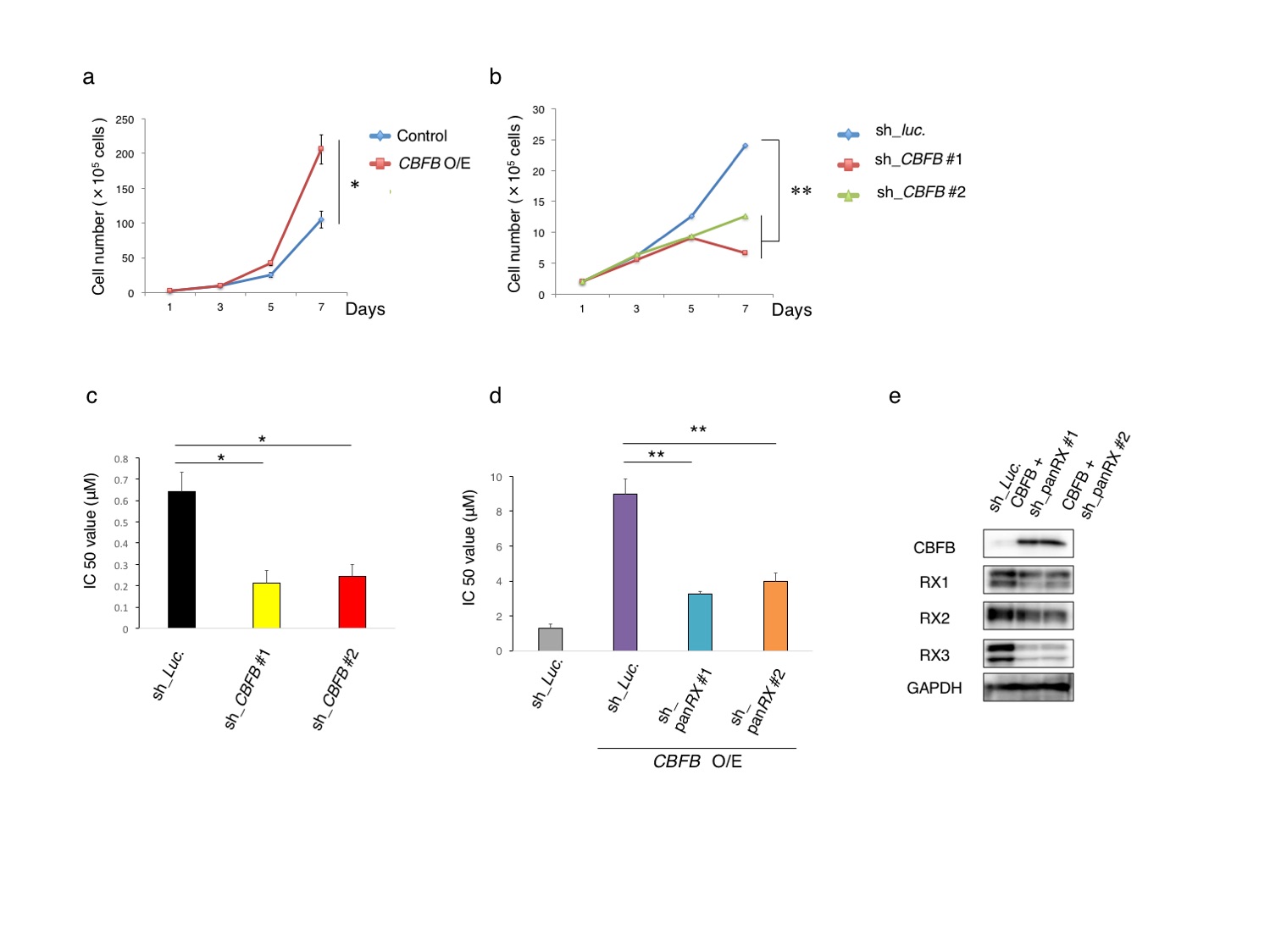
**

**Supplementary Fig. S5**

(a) CBFB overexpression confers proliferative advantage to AML cells. Growth curves of MV4-11 cells transduced with lentivirus encoding *CBFB* or control in the presence of 3 μM doxycycline up to 7 days (n = 3).

(b) CBFB down-regulation confers growth suppression to AML cells. Growth curves of MV4-11 cells transduced with control (sh_*Luc*.) or with *CBFB* shRNAs (sh_*CBFB* #1 and sh_*CBFB* #2) in the presence of 3 μM doxycycline up to 7 days (n = 3).

(c) IC50 values of Ara-C in CBFB-knocked down MV4-11 cells. MV4-11 cells were transduced with control (sh_*Luc*.) or with *CBFB* shRNAs (sh_*CBFB* #1 and sh_*CBFB* #2) and maintained in the presence of 3 μM doxycycline. Cells were then treated with various concentrations of Ara-C. Forty-eight hours after treatment, IC50 values were calculated (n = 3).

(d) CBFB-mediated Ara-C resistance is dependent on RUNX functions. MV4-11 cells were transduced with control (sh_*Luc*.) or with shRNAs targeting whole RUNX family members (sh_*panRX* #1 and sh_*panRX* #2) together with CBFB-expressing lentivirus and maintained in the presence of 3 μM doxycycline. Cells were then treated with various concentrations of Ara-C. Forty-eight hours after treatment, IC50 values were calculated (n = 3).

(e) RUNX family expressions in MV4-11 cells used in (d). Cells were treated with 3 μM doxycycline for 48 hours. After the treatment, cell lysates were analyzed by immunoblotting with the indicated antibodies. GAPDH was used as a loading control.

Data are mean ± SEM. * P < 0.05, ** P < 0.01, by two-tailed Student’s t-test.

**
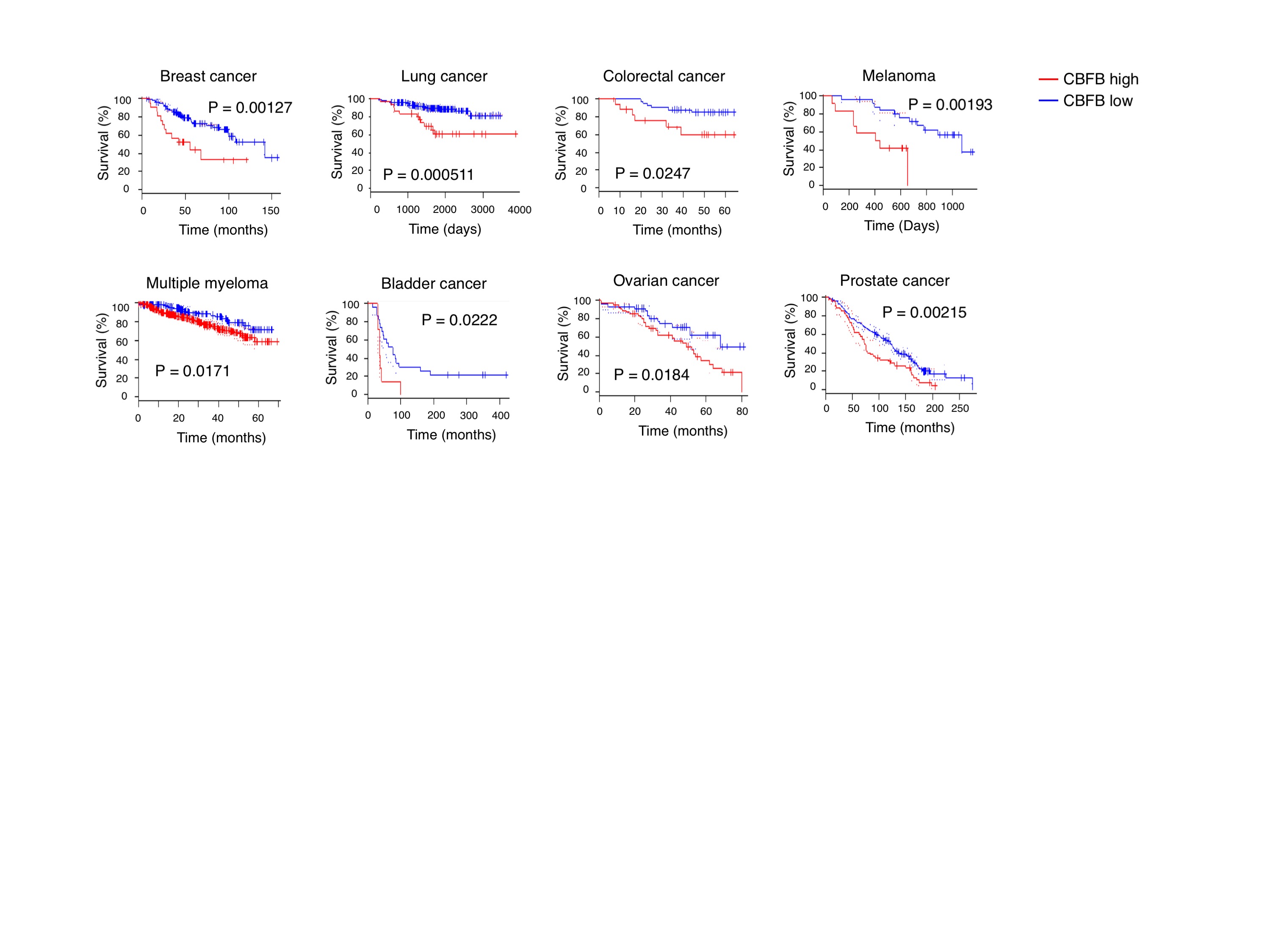
**

**Supplementary Fig. S6**

Overall survival of cancer patients with a higher or a lower expression level of *CBFB*. Lung cancer (GSE31210) with a higher (n = 31) and a lower *CBFB* (n = 173) breast cancer (GSE3143) with a higher (n = 21) and a lower *CBFB* (n = 137), colorectal cancer (GSE12945) with a higher (n = 18) and a lower *CBFB* (n = 44), ovarian cancer (GSE26712) with a higher (n = 64) and lower *CBFB* (n = 46), multiple myeloma (GSE2658, high n = 408, low n = 151), bladder cancer (GSE5287, high n = 7, low n = 23), prostate cancer (GSE16560, high n = 55, low n = 226) and Melanoma (GSE19234, high n = 12, low n = 26).

**Supplementary Tables**

**Supplementary Table S1**

List of primers used for RT-qPCR experiments in this study.

**Supplementary Table S2**

List of primers used for ChIP assay in this study.

**Supplementary Table S3**

List of target sequences for shRNA-mediated knockdown experiments in this study.

**Supplementary Table S4**

List of primers used for NGS experiments in this study.
